# Barriers to COVID-19 vaccine surveillance: the issue of under-reporting adverse events

**DOI:** 10.4178/epih.e2023054

**Published:** 2023-06-07

**Authors:** Yunha Noh, Hwa Yeon Ko, Ju Hwan Kim, Dongwon Yoon, Young June Choe, Seung-Ah Choe, Jaehun Jung, Ju-Young Shin

**Affiliations:** 1 School of Pharmacy, Sungkyunkwan University, Suwon, Korea; 2 Department of Epidemiology, Biostatistics, and Occupational Health, McGill University, Montreal, QC, Canada; 3 Department of Pediatrics, Korea University Anam Hospital, Seoul, Korea; 4 Department of Preventive Medicine, Korea University College of Medicine, Seoul, Korea; 5 Division of Life Sciences, Korea University, Seoul, Korea; 6 Department of Preventive Medicine, Gachon University College of Medicine, Incheon, Korea; 7 Department of Biohealth Regulatory Science, Sungkyunkwan University, Suwon, Korea; 8 Department of Clinical Research Design & Evaluation, Samsung Advanced Institute for Health Sciences & Technology (SAIHST), Sungkyunkwan University, Seoul, Korea

**Keywords:** COVID-19, Pharmacovigilance, Cross-sectional survey, Vaccine

## Abstract

**OBJECTIVES:**

This study investigated the reporting rates of adverse events following immunization (AEFIs) to the spontaneous reporting system (SRS) and its predictors among individuals with AEFIs after coronavirus disease 2019 (COVID-19) vaccination.

**METHODS:**

A cross-sectional, web-based survey was conducted from December 2, 2021 to December 20, 2021, recruiting participants >14 days after completion of a primary COVID-19 vaccination series. Reporting rates were calculated by dividing the number of participants who reported AEFIs to the SRS by the total number of participants who experienced AEFIs. We estimated adjusted odds ratios (aORs) using multivariate logistic regression to determine factors associated with spontaneous AEFIs reporting.

**RESULTS:**

Among 2,993 participants, 90.9% and 88.7% experienced AEFIs after the first and second vaccine doses, respectively (reporting rates, 11.6 and 12.7%). Furthermore, 3.3% and 4.2% suffered moderate to severe AEFIs, respectively (reporting rates, 50.5 and 50.0%). Spontaneous reporting was more prevalent in female (aOR, 1.54; 95% confidence interval [CI], 1.31 to 1.81); those with moderate to severe AEFIs (aOR, 5.47; 95% CI, 4.45 to 6.73), comorbidities (aOR, 1.31; 95% CI, 1.09 to 1.57), a history of severe allergic reactions (aOR, 2.02; 95% CI, 1.47 to 2.77); and those who had received mRNA-1273 (aOR, 1.25; 95% CI, 1.05 to 1.49) or ChAdOx1 (aOR, 1.62; 95% CI, 1.15 to 2.30) vaccines versus BNT162b2. Reporting was less likely in older individuals (aOR, 0.98; 95% CI, 0.98 to 0.99 per 1-year age increment).

**CONCLUSIONS:**

Spontaneous reporting of AEFIs after COVID-19 vaccination was associated with younger age, female sex, moderate to severe AEFIs, comorbidities, history of allergic reactions, and vaccine type. AEFIs under-reporting should be considered when delivering information to the community and in public health decision-making.

## INTRODUCTION

The spontaneous reporting system (SRS) is a cornerstone of drug safety surveillance in the post-approval phase [1]. It detects early signals of new, rare, or serious drug adverse events in a large population which cannot be identified in randomized clinical trials due to short-term follow-up and the rarity of adverse events [2]. The SRS also informs hypotheses leading to further investigations or regulatory warnings [3]. Its role has recently become more prominent with the emergency-use approval of coronavirus disease 2019 (COVID-19) vaccines [4]. During the unprecedented pandemic, COVID-19 vaccines have been developed rapidly, raising extensive safety concerns among the public [5]. Since mistrust of vaccines has a negative effect on vaccination campaigns, misconceptions about vaccine safety issues should be addressed quickly based on robust pharmacovigilance [6]. In this respect, the SRS plays a key role in the surveillance of adverse events following immunization (AEFIs) against COVID-19 and contributes to strengthening public confidence in COVID-19 vaccination and health authorities [4]. In addition, data retrieved from the SRS have been leveraged to examine specific AEFIs associated with the COVID-19 vaccine [7,8]. In Korea, the SRS for COVID-19 vaccines has actively utilized mobile and web-based platforms, which implemented more intensive pharmacovigilance than the routine SRS system.

However, under-reporting remains a major challenge for the SRS, which receives reports for only a small fraction of actual AEFIs. A systematic review of 37 studies has shown that 94% of AEFIs are not reported to the SRS [9]. Furthermore, the degree of under-reporting varies considerably by the type of drug, the type and severity of AEFIs, and media attention [10]. Several studies have reported the prevalence of AEFIs after COVID-19 vaccination using the SRS or participant-reported data [11,12]. However, there are limited data on the reporting rate of COVID-19 vaccines among individuals who actually experienced AEFIs after COVID-19 vaccination, although the rate of AEFIs reported to the SRS is important for evaluating vaccine safety signals properly. As the COVID-19 pandemic and vaccination against COVID-19 are expected to continue, it is important to understand the spontaneous reporting rate of AEFIs after COVID-19 vaccination to the SRS. Thus, in this study, we estimated the reporting rate of AEFIs to the SRS among individuals with AEFIs after COVID-19 vaccination, evaluated potential predictors of spontaneous reporting of AEFIs, and investigated the reasons for not reporting AEFIs in the Korean adult population.

## MATERIALS AND METHODS

### Participants

We conducted a cross-sectional, web-based survey from December 2, 2021 to December 20, 2021. We recruited participants aged 18-49 years > 14 days after they had completed a primary series of COVID-19 vaccination in Korea (i.e., those receiving either 2 doses of BNT162b2 [Pfizer-BioNTech], mRNA-1273 [Moderna], or ChAdOx1 [AstraZeneca], or a single dose of Ad26. COV2.S [J&J-Janssen]). The survey was circulated via email to Gallup panels stratified by age, sex, and region (nationally representative) using a proportional allocation method [13]. Of 22,790 invitations, 27.9% started the survey, and 67.9% of them finished it (Supplementary Material 1). We enrolled 2,993 respondents and weighted them by age, sex, and region to represent the Korean population. The total number of the weighted respondents was identical to that of the unweighted respondents.

### Variable measurements

Our measures were the prevalence of AEFIs after COVID-19 vaccination (calculated by dividing the number of individuals who reported experiencing AEFIs in our survey by the total number of participants) and the reporting rate of AEFIs to the SRS (calculated by dividing the number of participants who reported AEFIs to the SRS by the number of individuals who reported experiencing AEFIs in our survey). We asked all participants, “Did you experience any adverse events after the first (or second) dose of COVID-19 vaccine? Please check all that apply,” with 19 response options, including 17 types of predefined COVID-19 vaccine-related AEFIs, “other unsolicited symptoms” that could be responded to by free text, and “no AEFIs” (Supplementary Material 2). Those who reported experiencing any AEFIs against COVID-19 were asked, “After experiencing an AEFIs after the first (or second) dose of COVID-19 vaccine, have you reported the AEFIs to the SRS, including health professionals, pharmaceutical companies, the Korea Disease Control and Prevention Agency, etc.?” with response options of “yes” or “no.” If participants answered “no,” they were asked, “What is the primary reason you did not report the AEFIs after COVID-19 vaccination?” and prompted to choose 1 main reason from a list of 6 possible responses (Supplementary Material 2).

We predefined 17 AEFIs by referring to adverse reactions reported in clinical trials and described in package inserts of the vaccines [14,15]. Most of the predefined AEFIs are familiar as adverse reactions following the administration of other vaccines, and they have been mentioned in the vaccine safety surveillance guidelines of the World Health Organization [4] or the list of the monitoring system of the Korea Disease Control and Prevention Agency [16]. In addition, we included AEFIs that should be considered as potential signals of previously unrecognized vaccine-related adverse events (e.g., menstrual disorders or vaginal bleeding, mental illness) based on several case reports [17,18].

We assessed the following factors, selected based on expert knowledge and existing literature, as established or potential predictors of AEFIs reporting after COVID-19 vaccination: age, sex, region (categorized as urban and rural), education levels (no high school degree, high school graduate, undergraduate, and graduate degree), employment status, history of comorbidities, vaccination period, number of vaccination (first and second dose), type of COVID-19 vaccine received (BNT162b2, mRNA-1273, ChAdOx1, and Ad26.COV2.S), history of moderate to severe AEFIs after COVID-19 vaccination (defined in this study as AEFIs requiring hospital visits or admission), history of a severe allergic reaction, and anticoagulant use in the past six months. The information on comorbidities was collected based on 14 response options, including 12 types of medical conditions (diabetes, hypertension, heart diseases, cerebrovascular diseases, cancer, autoimmune diseases, skin diseases, respiratory diseases, renal diseases, liver diseases, dementia or other neurological diseases, and psychiatric diseases or mood disorders), “other,” which could be responded to by free text, and “not applicable” (Supplementary Material 2). These comorbidities were selected as major disorders that could affect the incidence and reporting rates of AEFIs after COVID-19 vaccination, including immunocompromised conditions and several chronic diseases, which are prioritized for COVID-19 vaccination in Korea. We hypothesized that individuals with any of these comorbidities would pay more attention to health conditions related to COVID-19 vaccination and were more likely to report AEFIs. We also selected anticoagulant use as a predictor because anticoagulant users were considered a high-risk group for adverse events such as bleeding or bruising after COVID-19 vaccination, which may have led them to have more vaccine safety concerns and an increased willingness to report AEFIs.

### Statistical analysis

The prevalence and reporting rate of AEFIs were described using frequency (percentage). To assess predictors of AEFIs reporting, we estimated odds ratios (ORs) with 95% confidence intervals (CIs) using univariate and multivariate logistic regression models conditional on the variables listed above. The unit of analysis was individuals with AEFIs. CIs not overlapping 1.0 were considered statistically meaningful. All statistical analyses were performed using SAS version 9.4 (SAS Institute Inc., Cary, NC, USA) and Microsoft Excel (Microsoft Corp., Redmond, WA, USA).

### Ethics statement

This study was conducted according to the Strengthening the Reporting of Observational studies in Epidemiology guidelines [19] and was approved by the Institutional Review Board of Sungkyunkwan University (SKKU 2021-11-019). All participants provided informed consent for the survey and had the option to exit or continue the survey after reading the informed consent statement, and patients’ information was stored in an anonymized structured format.

## RESULTS

**Figure 1.**
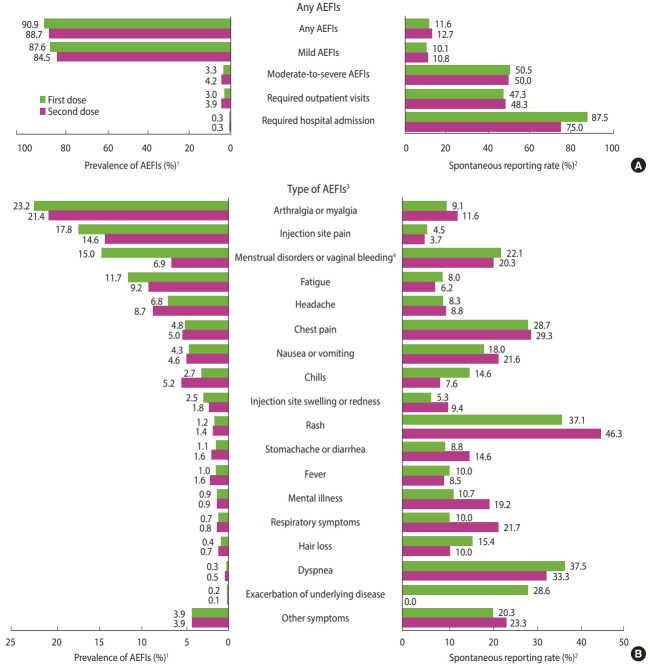
Spontaneous reporting rates of adverse events following immunization (AEFIs) against coronavirus disease 2019 (COVID-19). (A) Represents prevalence and spontaneous reporting rate of (A) any AEFIs and (B) each type of AEFIs. ^1^The percentage was calculated by dividing the number of individuals who reported experiencing AEFIs in our survey by the total number of participants. ^2^The percentage was calculated by dividing the number of individuals who reported AEFIs to the spontaneous reporting system by the number of those who reported experiencing adverse events in our survey. ^3^Descending by frequency of adverse events reported in this survey. ^4^The denominator is the number of female respondents (n=1,449).

Among 2,993 respondents (mean ± standard deviation age 34.6± 8.9 years, 48.4% female), 2,720 (90.9%) and 2,656 (88.7%) responded that they had experienced an AEFIs after the first and second vaccine dose, respectively; however, their reporting rates were only 11.6% (n=315) and 12.7% (n=337) (Figure 1). The majority of participants suffered mild AEFIs (87.6%, n =2,621 and 84.5%, n=2,530 after the first and second doses, respectively). Of the total participants, 3.3% (n=99) and 4.2% (n=126) suffered moderate to severe AEFIs that required hospital visits or admission after the first and second doses; their reporting rates were 50.5% (n=50) and 50.0% (n=63), respectively. The most common AEFIs were arthralgia or myalgia, injection site pain, and menstrual disorders or vaginal bleeding, whereas the reporting rates were the highest for rash, dyspnea, and chest pain.

**Figure 2.**
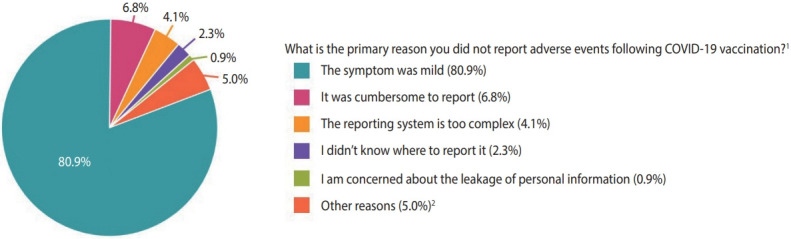
Reasons for not reporting adverse events following immunization against coronavirus disease 2019 (COVID-19). ^1^The answers include both first and second doses (n=4,724). ^2^Other reasons: “The adverse event was well-known” (1.2%) and “Reporting is useless because there is no action or compensation” (0.8%).

The spontaneous reporting rates of AEFIs were higher in females (adjusted odds ratio [aOR], 1.54; 95% CI, 1.31 to 1.81), participants with comorbidities (aOR, 1.31; 95% CI, 1.09 to 1.57), those with moderate to severe AEFIs (aOR, 5.47; 95% CI, 4.45 to 6.73), those with a history of a severe allergic reaction (aOR, 2.02; 95% CI, 1.47 to 2.77), and those who received mRNA-1273 and ChAdOx1 vaccines (aOR, 1.25; 95% CI, 1.05 to 1.49 and aOR, 1.62; 95% CI, 1.15 to 2.30), respectively, vs. BNT162b2), whereas the rate was lower in older individuals (aOR, 0.98; 95% CI, 0.98 to 0.99 per 1-year increase of age) (Table 1). The primary reasons for not reporting the AEFIs were “mild symptoms” (80.9%), followed by “cumbersomeness of reporting” (6.8%) and “complexity of the reporting system” (4.1%) (Figure 2).

## DISCUSSION

This survey-based study estimated the spontaneous reporting rate of AEFIs after COVID-19 vaccination (approximately 10% of mild AEFIs reported, and 50% of moderate to severe AEFIs) and identified several factors associated with spontaneous reporting, which included being of younger age, being female, experiencing moderate to severe AEFIs after COVID-19 vaccination, having 1 or more comorbidities, having a history of a severe allergic reaction, and receiving a specific type of COVID-19 vaccine.

The most prevalent AEFIs, such as arthralgia or myalgia, injection site pain, fatigue, and headache, are physiological manifestations of the inflammatory response to vaccination [20] and are already well known to occur following vaccination. However, about 15% of the female respondents in our survey reported menstrual disorders or vaginal bleeding, and about 0.9% reported experiencing mental illness after their first vaccination dose with no established biological mechanism to explain it. One study suggested plausible mechanisms such as immunological interference with the hormones driving the menstrual cycle or the effect on immune cells lining the uterus after vaccination [21]. Regarding adverse psychiatric reactions, one possible mechanism resulting in a psychotic state includes cytokine storm activity and N-methyl-D-aspartate receptor hypofunction elicited by vaccination [22]. The reported AEFIs data should be monitored for potential signals, and further investigations should be supported to establish whether causality exists.

Our results showed that AEFIs were reported more frequently by young participants. The majority of AEFIs after COVID-19 vaccination were gathered via mobile and web-based platforms in Korea; thus, older people may have had more of a barrier to response using those platforms than younger people [23]. Furthermore, females were more likely to report AEFIs than males. This can be explained in part by females stronger innate and adaptive immune responses, which may eventually have contributed to increased susceptibility to vaccination [24]. High reporting rates of AEFIs in females also could be explained by different attitudes towards AEFIs by sex [25]. A survey showed that females perceive disease more seriously and feel less indifferent toward their condition than males [26]. Thus, females might perceive AEFIs as more severe, resulting in a higher reporting rate. The high reporting rates for menstrual disorders or vaginal bleeding also reflect female-specific AEFIs. Similarly, respondents with comorbidities or a history of allergic reactions reported AEFIs more frequently in our survey. This finding suggests that health concerns stemming from painful experiences may provoke more attention to individuals’ post-vaccine conditions [27]. Besides, moderate to severe AEFIs, a key predictor of AEFIs reporting, have been shown to occur more frequently in females, younger people, and those with a medical history, resulting in higher reporting rates [28,29].

The type of vaccine received may also have influenced reporting rates. The different reporting rates per vaccine type may be affected by various factors, including individuals’ perception or preference for a specific vaccine, known safety issues or media attention, and the experience of severe or specific AEFIs according to vaccine types. In our survey, those who received mRNA-1273, ChAdOx1, and Ad26.COV2.S were more likely to report AEFIs than those who received BNT162b2, and this result is in line with another study comparing reporting rates of AEFIs across 4 different types of COVID-19 vaccines in the Netherlands [30]. This can be explained by a stronger preference for BNT162b2 over other vaccines [31], as well as differences in the occurrence of moderate to severe AEFIs [11,30]. Moreover, during the unprecedented pandemic, most people received vaccine types that they were assigned [32]. Thus, those who received undesired vaccine types or were worried about the safety of vaccines with a potentially higher risk of AEFIs may have been more willing to report their AEFIs to the SRS.

Our study provided primary data on the reporting rates of the SRS and its predictors following COVID-19 vaccination. The implication of this study will be significant over time as the COVID-19 pandemic continues, with the emergence of further variants, and several COVID-19 vaccines will be newly approved and used in public health. Our study also has some limitations. First, as capabilities and cultures for spontaneous reporting of AEFIs differ among countries, the reporting rate estimates can vary by country. Second, recall bias is possible, given the nature of the survey and the time gap between vaccination and survey response. Third, given the nature of a cross-sectional study, establishing causal relationships between AEFIs and vaccines could not be supported by our survey results.

In conclusion, the spontaneous reporting rate of AEFIs after COVID-19 vaccination was low, but most AEFIs were mild, while moderate to severe AEFIs were more likely to be spontaneously reported. Younger age, female sex, moderate to severe AEFIs, comorbidities, a history of allergic reactions, and vaccine type were associated with the reporting rate. The under-reporting of AEFIs should be considered when interpreting safety signals, delivering information to residents, and using the data in public health decision-making. Although the SRS plays a crucial role in the surveillance of AEFIs after COVID-19 vaccination by providing safety signals in a timely manner, it is not possible to provide accurate estimates of the rate of AEFIs and to assess a causal relationship between vaccination and adverse events. Thus, we acknowledge that these data are used mainly to find safety signals, and further in-depth studies are needed to elucidate the causality.

## DATA AVAILABILITY

Due to the nature of this study, participants in this study did not agree for their data to be shared publicly. The data that support the findings of this study can be provided by the principal investigator (J-Y Shin) upon reasonable request.

## Figures and Tables

**Table 1.** Factors associated with spontaneous reporting of AEFIs against COVID-19 to the surveillance system

Characteristics		Reported (n=652)	Unreported (n=4,724)	Crude	Adjusted
Age, mean±SD (yr)		33.8±8.7	34.9±8.9	0.99 (0.98, 0.996)	0.98 (0.98, 0.99)
Sex					
	Male	261 (40.0)	2,423 (51.3)	1.00 (reference)	1.00 (reference)
	Female	391 (60.0)	2,301 (48.7)	1.53 (1.31, 1.79)	1.54 (1.31, 1.81)
Region					
	Urban	462 (70.9)	3,466 (73.4)	1.00 (reference)	-
	Rural	190 (29.1)	1,258 (26.6)	1.12 (0.95, 1.33)	-
Education level					
	No high school degree	13 (2.0)	93 (2.0)	1.00 (reference)	-
	High school graduate	127 (19.5)	900 (19.1)	1.01 (0.57, 1.79)	-
	Undergraduate degree	436 (66.9)	3,212 (68.0)	0.97 (0.56, 1.69)	-
	Graduate degree	76 (11.7)	519 (11.0)	1.04 (0.58, 1.87)	-
Employment					
	Employed	199 (30.5)	1,330 (28.2)	1.00 (reference)	-
	Housemaker/unemployed	453 (69.5)	3,394 (71.8)	1.11 (0.94, 1.31)	-
Comorbid conditions					
	Yes	178 (27.3)	985 (20.9)	1.39 (1.17, 1.66)	1.31 (1.09, 1.57)
	No	474 (72.7)	3,739 (79.1)	1.00 (reference)	1.00 (reference)
Vaccination period					
	Feb-May 2021	33 (5.1)	208 (4.4)	1.00 (reference)	-
	Jun-Aug 2021	184 (28.2)	1,502 (31.8)	0.79 (0.54, 1.14)	-
	Sep-Nov 2021	435 (66.7)	3,014 (63.8)	0.92 (0.64, 1.30)	-
Number of vaccinations					
	First dose	315 (48.3)	2,405 (50.9)	1.00 (reference)	-
	Second dose	337 (51.7)	2,319 (49.1)	1.10 (0.95, 1.29)	-
Type of COVID-19 vaccine					
	BNT162b2	420 (64.4)	3,291 (69.7)	1.00 (reference)	1.00 (reference)
	mRNA-1273	181 (27.8)	1,116 (23.6)	1.25 (1.05, 1.49)	1.25 (1.05, 1.49)
	ChAdOx1	36 (5.5)	200 (4.2)	1.38 (0.98, 1.94)	1.62 (1.15, 2.30)
	Ad26.COV2.S	15 (2.3)	117 (2.5)	1.02 (0.61, 1.70)	1.31 (0.78, 2.21)
Moderate to severe AEFIs after COVID-19		113 (17.3)	112 (2.4)	6.13 (5.00, 7.52)	5.47 (4.45, 6.73)
History of a severe allergic reaction		44 (6.7)	122 (2.6)	2.46 (1.81, 3.34)	2.02 (1.47, 2.77)
Anticoagulant use in the past 6 mo		13 (2.0)	49 (1.0)	1.84 (1.06, 3.18)	1.17 (0.67, 2.06)

Values are presented as number (%) or odds ratio (95% confidence interval); Confidence intervals not overlapping 1.0 were considered statistically meaning full. AEFIs, adverse events following immunization; COVID-19, coronavirus disease 2019; SD, standard deviation.

## SUPPLEMENTARY MATERIALS

Supplementary Material 1. Survey details

Supplementary Material 2 Study questionnaires
