# Supplementary material for: Barriers to COVID-19 vaccine surveillance: the issue of under-reporting adverse events
Source: Epidemiol Health. 2023 Jun 7;45:e2023054. doi: 10.4178/epih.e2023054 (PMC10593585; doi:10.4178/epih.e2023054)
Supplement: Supplementary Material 1. — Survey details [file epih-45-e2023054-Supplementary-1.docx]

**Supplementary Material 1.** Survey details

| Period | Number of invitations | Number of contacted respondents | Number of not eligible respondents | Quota filled | Number of respondents interviewed successfully^*^ | Contact rate 2^†^ | Response rate 3^†^ | Cooperation rate 1^†^ |
| --- | --- | --- | --- | --- | --- | --- | --- | --- |
| December 2-20, 2021 | 22,790 | 6,348 | 1,330 | 613 | 2,993 | 27.9% | 18.9% | 67.9% |

^*^The survey did not allow for a partial interview.

^†^Calculated according to the American Association of Public Opinion Research (AAPOR) criterion [Available at: <https://www.aapor.org/Education-Resources/For-Researchers/Poll-Survey-FAQ/Response-Rates-An-Overview.aspx>].
