# Supplementary material for: Barriers to COVID-19 vaccine surveillance: the issue of under-reporting adverse events
Source: Epidemiol Health. 2023 Jun 7;45:e2023054. doi: 10.4178/epih.e2023054 (PMC10593585; doi:10.4178/epih.e2023054)
Supplement: Supplementary Material 2 — Study questionnaires [file epih-45-e2023054-Supplementary-2.docx]

**Supplementary Material 2.** Study questionnaires

Our study used data collected from 12 questionnaires focusing on the AE experienced following COVID-19 vaccination, and whether the survey participants reported the AEs in the surveillance system.

**Participants’ demographic characteristics**

**1. What is your age (in years)? ____**

**2. What is your gender?**

① Woman ② Man

**3. Where do you currently live?**

① Seoul ② Busan ③ Daegu ④ Incheon ⑤ Gwangju ⑥ Daejeon ⑦ Ulsan ⑧ Sejong ⑨ Gyeonggi

⑩ Gangwon ⑪ Chungbuk ⑫ Chungnam ⑬ Jeonbuk ⑭ Jeonnam ⑮ Gyeongbuk ⑯ Gyeongnam ⑰ Jeju

*※ ① ~ ⑨ was classified as “Urban” area, and ⑩ ~ ⑰ as “Rural” area.*

**4. What is your highest level of education?**

**(e.g., If you drop out of high school, respond to ‘③ middle school graduation’)**

① No education ② Elementary school graduation ③ Middle school graduation ④ High school graduation

⑤ 2~3 year Bachelor’s degree ⑥ 4~6 year Bachelor’s degree ⑦ Graduate school or above

**5. Which of the following is your occupation?**

① Managers ② Professionals ③ Technicians and associate professionals ④ Clerical support workers

⑤ Service and sales workers ⑥ Skilled agricultural, forestry and fishery workers ⑦ Craft and related trades workers

⑧ Plant and machine operators, and assemblers ⑨ Elementary occupations ⑩ Armed forces occupations

⑪ Housemaker or currently no other job

*※ ⑪ was classified as “Housemaker/Unemployed” and the others as “Employed.”*

**Participants’ clinical characteristics**

**6. Please check the medical conditions for which you are currently being treated (select all that apply).**

① Diabetes ② Hypertension ③ Heart diseases ④ Cerebrovascular diseases ⑤ Cancer ⑥ Autoimmune diseases ⑦ Skin diseases ⑧ Respiratory diseases ⑨ Renal diseases ⑩ Liver diseases ⑪ Dementia or other neurological diseases ⑫ Psychiatric diseases or mood disorders ⑬ Others (respond by free text: ___) ⑭ Not applicable

**7. Have you ever experienced a severe allergic reaction (e.g., anaphylaxis, systemic erythema/edema, syncope, etc.) from vaccines or medications before being vaccinated against COVID-19?**

① Have experienced a severe allergic reaction ② Not experienced

**8. Have you ever taken anticoagulants in the last six months?**

① Yes ② No

**COVID-19 vaccine information**

**9. Which of the following COVID-19 vaccines (manufacturer) did you receive?**

|  | AstraZeneca | Pfizer-BioNTech | Moderna | J&J-Janssen | Others |
| --- | --- | --- | --- | --- | --- |
| **First dose** | ① | ② | ③ | ④ | ⑤ |
| **Second dose** | ① | ② | ③ | ④ | ⑤ |

**10. When were you vaccinated against COVID-19?**

|  | February 2021 | March-May 2021 | June-August 2021 | September-November 2021 |
| --- | --- | --- | --- | --- |
| **First dose** | ① | ② | ③ | ④ |
| **Second dose** | ① | ② | ③ | ④ |

**The experience and reporting of adverse events following COVID-19 vaccination**

**11. Did you experience any adverse reactions or side effects after the first dose of the COVID-19 vaccine? Please check all that apply.**

(1) injection site pain (2) injection site swelling or redness (3) fever (4) fatigue (5) chills (6) headache

(7) arthralgia or myalgia (8) nausea or vomiting (9) stomachache or diarrhea (10) rash

(11) exacerbation of underlying disease (12) dyspnea (13) other respiratory symptoms (cough, etc.)

(14) mental illness (anxiety, depression, insomnia, aggravation of mental disorder, etc.) (15) Chest pain (16) hair loss (17) Menstrual disorders or vaginal bleeding (18) other unsolicited symptoms (respond by free text: ____)

**➡ Go to question 11-1**

(19) No adverse reaction **➡ Go to question 12**

**11-1. What was the severity of adverse events after the first dose of the COVID-19 vaccine?**

① Mild enough to not interfere with daily life

② Disruptive to daily life but not enough to visit a hospital

③ Required hospital visit but not enough to be hospitalized

④ Required hospital admission (including intensive care unit or emergency room)

**11-2. After experiencing adverse events after the first dose of COVID-19 vaccine, have you reported the adverse events to the spontaneous reporting system, including reporting to health professionals, pharmaceutical companies, Disease Control and Prevention Agency, etc.?**

① Yes **➡ Go to question 12** ② No **➡ Go to question 11-3**

**11-3. What is the primary reason you did not report adverse events following the COVID-19 vaccination?**

① The symptom was mild.

② It was cumbersome to report.

③ The reporting system is too complex.

④ I didn't know where to report it.

⑤ I am concerned about the leakage of personal information.

⑥ Other reasons (respond by free text: ____)

**12. Did you experience any adverse reactions or side effects after the second dose of the COVID-19 vaccine? Please check all that apply.**

(1) injection site pain (2) injection site swelling or redness (3) fever (4) fatigue (5) chills (6) headache

(7) arthralgia or myalgia (8) nausea or vomiting (9) stomachache or diarrhea (10) rash

(11) exacerbation of underlying disease (12) dyspnea (13) other respiratory symptoms (cough, etc.)

(14) mental illness (anxiety, depression, insomnia, aggravation of mental disorder, etc.) (15) Chest pain (16) hair loss (17) Menstrual disorders or vaginal bleeding (18) other unsolicited symptoms (respond by free text: ____)

**➡ Go to question 12-1**

(19) No adverse reaction **➡ End the survey**

**12-1. What was the severity of adverse events after the second dose of the COVID-19 vaccine?**

① Mild enough to not interfere with daily life

② Disruptive to daily life but not enough to visit a hospital

③ Required hospital visit but not enough to be hospitalized

④ Required hospital admission (including intensive care unit or emergency room)

**12-2. After experiencing adverse events after the second dose of COVID-19 vaccine, have you reported the adverse events to the spontaneous reporting system, including reporting to health professionals, pharmaceutical companies, Disease Control and Prevention Agency, etc.?**

① Yes **➡ End the survey** ② No **➡ Go to question 12-3**

**12-3. What is the primary reason you did not report adverse events following the COVID-19 vaccination?**

① The symptom was mild.

② It was cumbersome to report.

③ The reporting system is too complex.

④ I didn't know where to report it.

⑤ I am concerned about the leakage of personal information.

⑥ Other reasons (respond by free text: ____)
